# Secondary Complement Deficiency Impairs Anti-Microbial Immunity to *Klebsiella pneumoniae* and *Staphylococcus aureus* During Severe Acute COVID-19

**DOI:** 10.3389/fimmu.2022.841759

**Published:** 2022-04-27

**Authors:** Youssif M. Ali, Nicholas J. Lynch, Priyanka Khatri, Ifeoluwa E. Bamigbola, Andrew C. Y. Chan, Munehisa Yabuki, Gregory A. Demopulos, Jonathan L. Heeney, Sumita Pai, Helen Baxendale, Wilhelm J. Schwaeble

**Affiliations:** ^1^ Department of Veterinary Medicine, School of Biological Sciences, University of Cambridge, Cambridge, United Kingdom; ^2^ Department of Microbiology and Immunology, Faculty of Pharmacy, Mansoura University, Mansoura, Egypt; ^3^ Omeros Corporation, Seattle, WA, United States; ^4^ Royal Papworth Hospital NHS Foundation Trust, Cambridge, United Kingdom

**Keywords:** *K. pneumoniae*, COVID-19, SARS-CoV-2, complement system, bacterial infection, *S. aureus*

## Abstract

A high incidence of secondary *Klebsiella pneumoniae* and *Staphylococcus aureus* infection were observed in patients with severe COVID-19. The cause of this predisposition to infection is unclear. Our data demonstrate consumption of complement in acute COVID-19 patients reflected by low levels of C3, C4, and loss of haemolytic activity. Given that the elimination of Gram-negative bacteria depends in part on complement-mediated lysis, we hypothesised that secondary hypocomplementaemia is rendering the antibody-dependent classical pathway activation inactive and compromises serum bactericidal activity (SBA). 217 patients with severe COVID-19 were studied. 142 patients suffered secondary bacterial infections. Klebsiella species were the most common Gram-negative organism, found in 58 patients, while *S. aureus* was the dominant Gram-positive organism found in 22 patients. Hypocomplementaemia was observed in patients with acute severe COVID-19 but not in convalescent survivors three months after discharge. Sera from patients with acute COVID-19 were unable to opsonise either *K. pneumoniae* or *S. aureus* and had impaired complement-mediated killing of Klebsiella. We conclude that hyperactivation of complement during acute COVID-19 leads to secondary hypocomplementaemia and predisposes to opportunistic infections.

## Introduction

Coronavirus disease 2019 (COVID-19), a predominantly respiratory disease caused by Severe Acute Respiratory Syndrome-Coronavirus type 2 (SARS-CoV-2), is responsible for the current global health pandemic, with a high rate of mortality, especially among the elderly and patients with underlying medical conditions (1). Secondary bacterial infections with different microbial pathogens such as *Streptococcus pneumoniae, Klebsiella pneumoniae, Haemophilus influenzae, Escherichia coli*, *Staphylococcus aureus*, and Aspergillus have been reported during COVID-19 (2). The mechanisms involved in the increased risk of secondary infection are likely multifactorial including known underlying risk factors for infection such as immune deficiency, gastric reflux/aspiration and gut ischaemia associated with severe disease, and the use of catheters and lines to support critical care management provides a portal for infection and sustained colonisation. Lung tissue injury through SARS-CoV-2 infection may facilitate bacterial colonisation, resulting in airway dysfunction, cytopathology, tissue destruction and damage to the protective mucosa in the lung, exacerbating disease severity and increasing the risk of septicaemia and admission to the intensive care unit (ICU) (3). In the acute inflammatory phase of severe COVID-19, a secondary innate and adaptive immune incompetence is likely to increase the risk of secondary infections. It is well established that sepsis can impair many aspects of immune functions (4) *K. pneumoniae* is a Gram-negative opportunistic pathogen that causes serious pathology such as pneumonia, septicaemia, urinary tract infection (UTI) and pyogenic liver abscesses (5). The incidence of Klebsiella infection is increasing, with the highest incidence in older age groups, as has recently been reported in England (https://assets.publishing.service.gov.uk/government/uploads/system/uploads/attachment_data/file/615375/hpr1817_klbsll.pdf).

The clinical management of secondary *K. pneumoniae* infections became a serious issue during the COVID-19 pandemic because *Klebsiella* strains (and possibly other opportunistic pathogens) have developed mechanisms to resist a wide range of antimicrobial agents, such as β-lactams, aminoglycosides, quinolones, and polymyxins (6). Although antibiotic treatment of patients infected with *K. pneumoniae* may reduce bacterial load, most antibiotics offer insufficient protection from organ damage resulting from an exaggerated immune response. *K. pneumoniae* produces a wide range of virulence factors, such as capsular polysaccharides and lipopolysaccharide (endotoxins), and leads to biofilm formation (mucoid layer), all of which increase the pathogenicity of the bacteria (7). The contribution of the mucoid layer to the pathogenicity of *K. pneumoniae* strains has been reported to increase the resistance to phagocytosis and serum killing activity by preventing direct complement activation on the bacterial surface (7). As such, an anti-capsular antibody is required to enable complement fixation and optimal bacterial clearance of mucoid strains. Mucoid strains of *K. pneumoniae* are normally responsible for invasive disease and community-acquired pneumonia, whereas non-mucoid strains of Klebsiella are less virulent (8).

*Staphylococcus aureus* is a Gram-positive opportunistic bacterium causing infections that vary from superficial skin infection to life-threatening invasive disease including pneumonia and sepsis (9). The transition from an opportunistic commensal to an invasive pathogen requires evasion from the immune defence and the ability of the bacterium to exploit different niches within the host. Secondary infections caused by *S. aureus* and methicillin-resistant *Staphylococcus aureus* (MRSA) are commonly feared, especially among immunocompromised and severely ill patients as they contribute to further morbidity and mortality (10). The increased risk for *S. aureus* infections during COVID-19 was reported in previous studies showing an association between secondary infections with *S. aureus* and MRSA and mortality (10, 11).

The complement system is a major component of innate immunity and plays a pivotal role in the prevention of invasive microbial infections (12). The complement activation cascade is initiated *via* three different pathways: the classical (CP), the lectin (LP), and the alternative (AP) pathways (13, 14). Initiation of complement activation converges in the generation of enzyme complexes that cleave the most abundant complement component C3, generating the activation fragments C3b and C3a. While C3a is an anaphylatoxin, C3b binds covalently to activating surfaces, like the surface of bacteria, to enhance their uptake and removal by phagocytic cells. C3b binds in close proximity of the C3 convertase complexes C3bBb and C4bC2a, switching their substrate specificity from C3 to C5. C5 is split into the anaphylatoxin C5a and the larger fragment C5b, which initiates the formation of the terminal pathway and results in the formation of C5b-9, ultimately leading to the insertion of this complex – the membrane attack complex (MAC) – in the cell wall, forming a channel-like pore composed of polymers of C9. Membrane penetrating C5b-C9 complexes cause osmotic leakage and lyse bacteria (15).

The first indication that complement is likely to be involved in the inflammatory pathology of severe acute respiratory syndrome coronavirus (SARS-CoV) infection was published before the emergence of SARS-CoV-2 in a mouse model of SARS-CoV-1 where gene-targeted C3-deficient mice were protected from the significant weight loss and respiratory dysfunction seen in C56BL/6J wildtype control mice infected with an equivalent viral load (16).

Following the emergence of SARS-CoV-2 in 2019, it became clear that some infected individuals can develop moderate, to severe, to life-threatening forms of COVID-19 presenting as an acute respiratory distress syndrome (ARDS), and an early histopathology study in the tissue of patients that succumbed to COVID-19 provided strong evidence of an intrinsic involvement of complement activation and microangiopathies in the pathophysiology of COVID-19 (17). Many articles have been published since reporting the involvement of complement in the pathophysiology of COVID-19, but how and through which initiation pathway a substantial activation of complement occurs is still elusive (18, 19).

Several previous studies reported the essential role of complement activation during *K. pneumoniae* and *S. aureus* (20–23). This report demonstrates that hyperactivation of complement seen in every COVID-19 patient serum assessed in the acute phase of severe disease leads to a secondary loss of complement-dependent opsonisation of *K. pneumoniae* and *S. aureus* and complement–mediated lysis of *K. pneumoniae*. The findings of this study provide an explanation for the frequent occurrence of opportunistic secondary infections with *K. pneumoniae and S. aureus* in patients with severe COVID-19.

## Materials and Methods

### Clinical Data and Serum Samples

SARS-CoV-2 infected patients referred to the Royal Papworth Hospital, Cambridge, UK for critical care were recruited to the study. Clinical assessment and WHO criteria scoring of severity was conducted following the ‘COVID-19 Clinical Management: living guidance’. (*COVID-19 Clinical Management: Living Guidance*. Available at: https://www.who.int/publications/i/item/WHO-2019-nCoV-clinical-2021-1. 217 patients included in our study were classified as severely ill (scoring between 3 and 7 in the WHO severity score, see above). Blood and sputum cultures were collected and processed using standard microbiological techniques as part of routine clinical care. Bacteria were identified according to UK standards for Microbiological investigations https://assets.publishing.service.gov.uk/government/uploads/system/uploads/attachment_data/file/800451/B_57i3.5.pdf. Serum samples were taken at defined time intervals from hospital admission up to convalescence from 25 severely ill patients (scoring between 4-7). In this 25-patient group, there are 10 females and 15 males with an average age of 51 years (ranging from 30-73 years). All critically ill patients are at risk of venous thromboembolism and dissemminated intravascular coagulation (DIC). A high incidence of venous and arterial embolism (25-30%) has recently been reported in COVID-19 patients (24, 25). Patients with the more severe form of COVID-19 pneumonia display high D-dimers, low antithrombin, high fibrinogen and sometimes abnormal prothrombin time and activated thromboplastin time consistent with DIC. To manage the risk of thrombosis, all patients in our cohort received anticoagulant treatment throughout ICU stay and hospitalisation (either infusion with heparin [4500 IU/day] for all patients on extracorporeal membrane oxygenation (ECMO) or with low-molecular-weight heparin [LMWH] at intermediate doses of 50-60mg/day. To assess the impact of therapeutic doses of heparin or LMWH in the patients’ sera as a limiting confounding factor that might affect complement functional activity, we compared normal human serum (NHS), heparinised plasma and EDTA plasma in a C3b or C4b deposition assay on *K. pneumoniae*-coated ELISA plates. In addition, the CH_50_ of NHS, heparinised plasma (10 IU heparin/mL) or EDTA plasma (10μM EDTA) was determined. No difference in complement functional activity was observed (**Figure S1**), an observation underlined by recent literature reporting that SBA against *K. pneumoniae* was not diminished in heparinised (5 IU/mL) blood (26).

The NHS control group is composed of sera from 6 females and 8 males with an average age of 47 years (ranging from 32 to 54 years). All NHS blood donors were tested negative for SARS-CoV-2 prior to blood collection.

The study was approved by Research Ethics Committee Wales, IRAS: 96194 12/WA/0148. Amendment 5. All participants or legal consent representatives provided written, informed consent prior to enrolment in the study. Sera from non-infected healthy volunteers were used as a control (NHS).

### Bacterial Strains

Mucoid (ATCC 43816) and non-mucoid (Ecl8) strains were kindly provided by Dr. Sebastian Bruchmann, Department of Veterinary Medicine, University of Cambridge, UK. ***Staphylococcus aureus*** (*S. aureus*) Newman strain D2C (ATCC^®^ 25904™) was purchased from ATCC.

### Measurement of Serum Levels of Complement Proteins and Complement Activation Products

Circulating C5a and sC5b-9 levels were measured using a sandwich ELISA kit supplied by R&D systems (Cat. No. DY2037) and (BD OptEIA Human C5b-9 ELISA set). Complement C3 and C4 levels were measured using Abcam C3 and C4 ELISA kits.

### Haemolytic Assay

2mL of packed sheep erythrocytes were washed 3 times using GVB buffer (10mM barbital, 145mM NaCl, 0.1%w/v bovine gelatine) containing 10mM EDTA. The final concentration of RBCs was adjusted to 1x10^9^/mL in the same buffer. RBCs were sensitised by incubation with 10μg/mL anti-sheep RBCs at 37°C with gentle shaking for 30 minutes. Finally, RBCs were washed with GVB buffer containing 2mM Ca^2+^ and 1mM Mg^2+^ (GVB^++^). Serum samples were serially diluted in GVB^++^ buffer in 96 well plates and 10^7^ RBCs were added to each well. Wells receiving water were used as a positive control to achieve 100% lysis of RBCs. Wells containing buffer only were used as a negative control. After 1 h incubation at 37°C, plates were centrifuged and released haemoglobin was measured at 405 nm. % RBCs haemolysis was calculated as previously described (27). In some experiments the haemolytic assay was performed using sera from acute COVID-19 patients reconstituted with purified human C4 (10μg/mL). C4 was purified from plasma given by healthy donors as previously described (28).

### Complement Activation Assay

Maxisorp polystyrene microtiter ELISA plates were coated with 10μg/mL mannan or formalin-fixed *K. pneumoniae* or *S. aureus* (OD600 = 0.6) in carbonate buffer (15mM Na_2_CO_3_, 35 mM NaHCO_3_, pH 9.6). The next day, wells were blocked with 1% BSA in TBS buffer (10mM Tris-HCl, 140mM NaCl, pH7.4) for 2 hours then washed with TBS buffer containing 0.05% (v/v) Tween 20 and 5 mM CaCl_2_. NHS were diluted in BBS^++^ buffer (4mM barbital, 145mM NaCl, 2mM CaCl_2_, 1 mM MgCl_2_, pH 7.4) (starting from 1:100), added to the plate and incubated for 1 hour at 37°C then washed. Deposition of C3b, C4b and C5b-9 was detected using either rabbit anti-C3c (Dako), rabbit anti-C4c (Dako) or rabbit anti-C5b-9 (Abcam), respectively, followed by peroxidase-conjugated goat anti-rabbit IgG. After 1 hour, wells were washed and 100 μL of 1-Step Ultra TMB Solution (Thermo Fisher Scientific) was then added to each well and incubated for 5 minutes at room temperature. The reaction was stopped by the addition of 2M H_2_SO_4_ and the optical density at 450 nm was immediately measured. To assess complement deposition *via* the alternative pathway, ELISA plates coated with *K. pneumoniae* and *S. aureus* were incubated with serial dilutions of NHS in EGTA buffer (4mM barbital, 145mM NaCl, 5mM MgCl_2_, 20mM EGTA, pH 7.4) starting from 1:5. The plate was incubated at 37°C for 1 hour then washed. C3b deposition was detected as described above.

### LP and CP Specific Complement Deposition Assay

To assess LP-mediated C4b deposition on the surface of *K. pneumoniae* and *S. aureus*, sera were diluted in MBL binding buffer (20mM Tris-HCl, 1 M NaCl, 10mM CaCl2, 0.05% (v/v) Triton X-100 pH 7.4) then incubated with ELISA plates coated with the bacteria for 1 hour at 37°C. After three washing steps, 100μL of 1μg/mL purified human C4 (Comp Tech, USA) in BBS^++^ was added to each well then incubated (29). After 1-hour incubation, plates were washed and bound C4b was detected as previously mentioned. To assess for CP activation, C1q-depleted serum (Comp Tech, USA) was serially diluted in BBS^++^ then incubated for 1 h at 37°C with ELISA plates coated with *K. pneumoniae*. As a control, C1q-depleted serum was reconstituted with 10μg/mL of purified human C1q. Complement C3b deposition was detected as described before. To inhibit the CP-mediated C3b deposition we used a potent monospecific anti-human C1s antibody (TNT003), which is a potent CP inhibitor (30). In this experiment, 2% NHS was incubated with different concentrations of monospecific anti-human C1s antibody (TNT003) at room temperature for 15 minutes then incubated with an ELISA plate coated with the bacteria for 15 minutes at 37°C. After several washing steps, complement C3b deposition was detected as described before.

### Complement Deposition From Acute and Convalescent COVID-19 Patients’ Sera on *K. pneumoniae* and *S. aureus*

ELISA plates coated with *K. pneumoniae* and *S. aureus* were incubated at 37°C with sera from acutely ill and convalescent COVID-19 patients (diluted 1:100) in BBS^++^. NHS was used as a control. After 1 hour, plates were washed and C3b, C4b or C5b-9 were detected using either rabbit anti-C3c, rabbit anti-C4c or rabbit anti-C5b-9, respectively, followed by peroxidase-conjugated goat anti-rabbit IgG as described before (31).

### Serum Bactericidal Assay (SBA)

*K. pneumoniae* isolates were grown in nutrient broth at 37°C for overnight with gentle shaking. The next day, 10 mL of fresh nutrient broth were seeded with 100 μL of overnight bacterial culture and incubated at 37°C with gentle shaking until mid-logarithmic phase. Bacterial cultures were collected, washed twice using BBS^++^ and then adjusted to a final concentration of 1×10^7^ CFU mL−1. 1×10^4^ CFU were incubated with 75% serum NHS or sera from acute/convalescent in BBS at 37°C with gentle shaking. After 2 hours, samples were taken and plated out on nutrient agar plates then incubated overnight at 37°C. Serum bactericidal activity was calculated by measuring the decrease in the viable bacterial count after 2 h incubation with each serum compared to heat-inactivated normal human serum (HI-NHS) (32).

### Determination of Antibody Titer Against *K. pneumoniae* in Patients’ Sera

Nunc Maxisorp microplates coated with *K. pneumoniae* and blocked using 1% BSA in TBS buffer as described above were used in this experiment. Wells were incubated at room temperature with different serum dilutions from SARS-CoV-2 or convalescent patients. Sera from non-COVID-19 volunteers were used as controls. After 1 h incubation, wells were washed with wash buffer and 100μL of peroxidase-conjugated goat anti-human IgG were added to each well. After 1 h, wells were washed and 100μL of 1-Step Ultra TMB Solution (Thermo Fisher Scientific) was then added to each well and incubated for 5 minutes at room temperature. The reaction was stopped by the addition of 2 M H_2_SO_4_ and the optical density at 450 nm was immediately measured. Antibody titre was calculated as the highest serum dilution that gave positive results (33).

## Results

### *K. pneumoniae* and *S. aureus* Secondary Bacterial Infections Are High Among COVID-19 Patients

**Figure 1:**
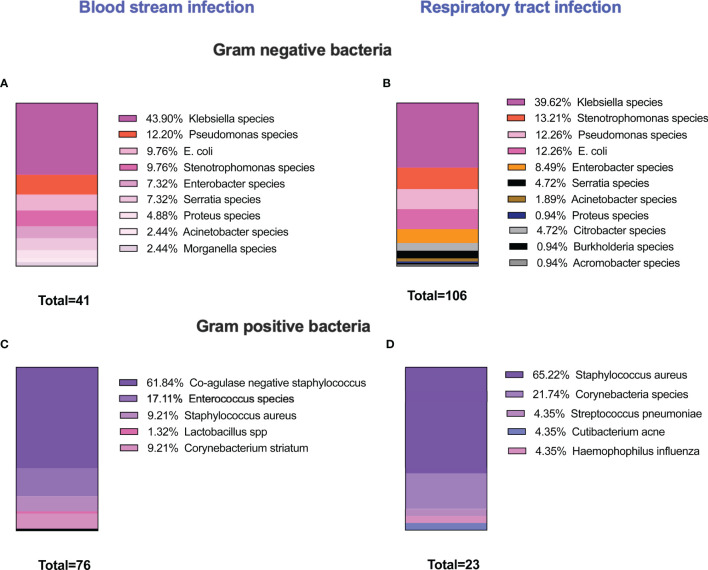
Prevalence of secondary bacterial infection among severely ill COVID-19 patients. 217 patients were recruited in this study. 142 patients developed secondary bacterial infections. The microbiology analysis showed that different microbial species were isolated either from blood or respiratory cultures. The majority of infections were caused by Gram-negative bacteria, and *Klebsiella* species were the most common organism detected either in blood or respiratory cultures **(A, B)**. High incidences of Gram-positive bacteria were also identified and coagulase-negative staphylococcus species were the dominant Gram-positive bacteria in blood cultures, and *S. aureus* was the predominant organism in respiratory cultures **(C, D)**. In several occasions, multiple microorganisms were isolated from one patient.

An unusually high rate of secondary bacterial infection was observed in patients admitted with severe symptoms of COVID-19 infection. Our analysis showed that, in our cohort of 217 severely ill COVID-19 patients, 142 presented with a secondary bacterial infection. A wide range of pathogens was isolated from sputum, BAL or blood, and the most common Gram-negative bacterial isolates were *Klebsiella* species (**Figures 1A, B**). Gram-positive bacteria were also identified in blood and respiratory cultures where *Staphylococcus* species were the dominant bacteria (**Figures 1C, D**).

### *K. pneumoniae* and *S. aureus* Activate Complement *via* the LP, CP, and the AP

**Figure 2:**
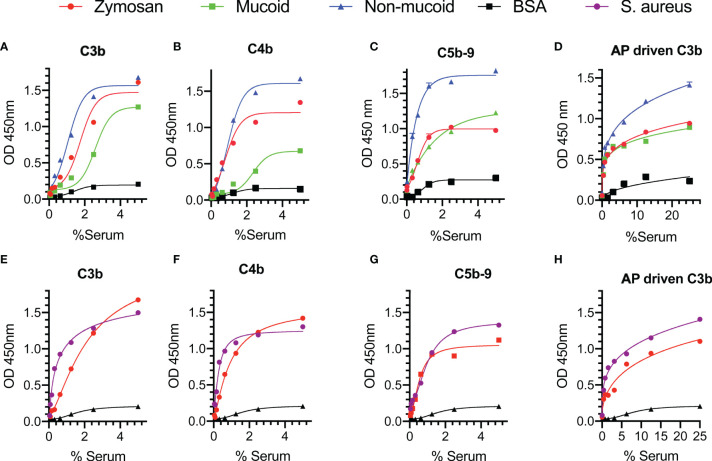
Complement is activated on the surface of *Klebsiella pneumoniae* and *Staphylococcus aureus*. ELISA plates were coated with mucoid or non-mucoid strains of *K. pneumoniae* or with *S. aureus*. Wells coated with zymosan were used as a control. Plates were incubated with NHS in BBS with Ca^+2^ and Mg^+2^ **(A–C, E–G)** or EGTA buffer **(D, H)**. Complement C3b, C4b and C5b-9 deposition were detected using specific antibodies. C3b, C4b and C5b-9 deposition were observed on the surface of *K. pneumoniae* **(A–C)** and *S. aureus* **(E–G)** in conditions permissive for both the CP and the LP pathways. High levels of C3b *via* the AP were also detected on the surface of the bacteria **(D, H)**. Results are means of duplicates ± SD.

In a set of preliminary experiments, we assessed complement deposition on the surface of mucoid and non-mucoid strains of *K. pneumoniae* using pooled sera from healthy volunteers. High levels of C3b, C4b and C5b-9 deposition were observed on the surface of the bacteria under conditions that allow activation of both the LP and CP, i.e., where serum was diluted in BBS with Ca^+2^ and Mg^+2^ (**Figures 2A**–**C**). Activation of the complement system was also observed on the surface of *S. aureus* where high levels of complement C3b, C4b and C5b-9 deposition were detected (**Figures 2E–G**).

Involvement of the AP in complement-mediated opsonisation was assessed by measuring C3b deposition under conditions that allow only activation of the AP, i.e., where serum samples were diluted in EGTA buffer with Mg^+2^. High levels of C3b deposition *via* the AP were detected on both *K. pneumoniae* and *S. aureus* (**Figures 2D, H**).

**Figure 3:**
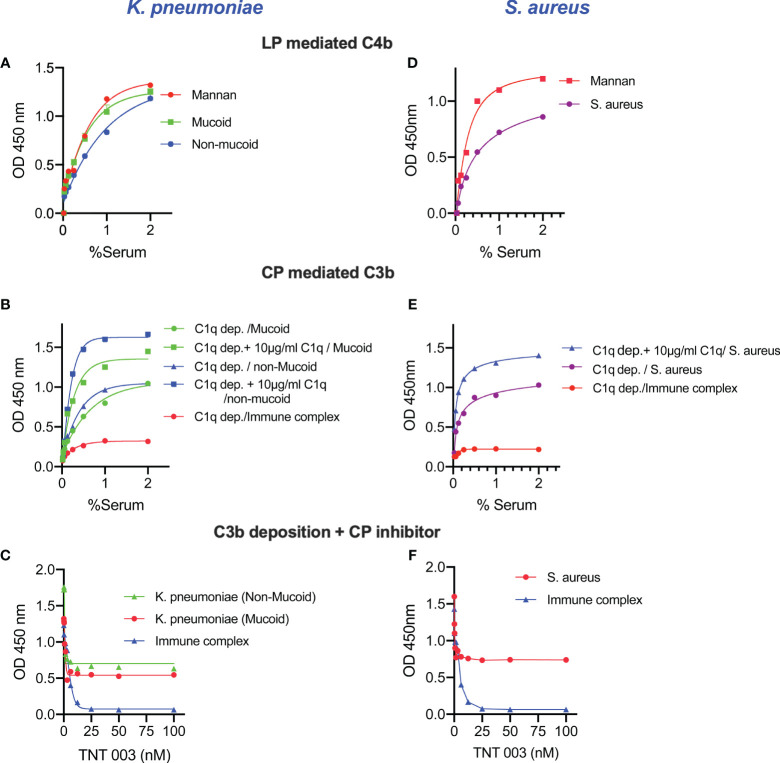
Complement activation pathway-specific assay conditions demonstrate that both LP and CP are activated on the surface of *K. pneumoniae* and *S. aureus*. ELISA plates coated with zymosan (as control), mucoid, non-mucoid strains of *K. pneumoniae* or *S. aureus* were incubated with NHS diluted in MBL-binding buffer for 1 h at 37°C then washed. Purified human C4 was incubated with the ELISA plate for another 1 h at 37°C. After washing steps, C4b deposition was detected. High levels of LP-mediated C4b deposition were observed in the surface of *K. pneumoniae* and *S. aureus* **(A, D)**. Likewise, the use of C1q-depleted serum resulted in a significant reduction of C3b deposition. In absence of CP functional activity, only LP-dependent C3b deposition was measured on the surface of *K.pneumoniae* and *S. aureus*. Reconstitution of C1q-depleted serum with purified human C1q restored CP-mediated C3b deposition. No C3b deposition was observed when using C1q-depleted serum on the surface of immune complexes **(B, E)**. Using the C1s inhibitory antibody TNT003 blocked CP-mediated C3b deposition on the surface of immune complexes and all CP-mediated C3b opsonisation on *K. pneumoniae* and *S. aureus*. Under the chosen assay conditions, the residual deposition of C3b on *Klebsiella* and *S. aureus* is LP-mediated **(C, F)**. Results are means of duplicates ± SD.

To evaluate complement activation on the surface of the bacteria by either the LP or the CP we used pathway-specific assay conditions. Sera diluted in MBL-binding buffer were incubated on ELISA plates coated with *K. pneumoniae* or *S. aureus* to measure the LP-dependent deposition of C4b (**Figures 3A, D**). The high salt content of the MBL-binding buffer dissociates the CP initiation complex C1 while it leaves the LP-initiation complexes intact (29). In an additional series of experiments, the contribution of the LP towards the deposition of C3b on the surface of bacteria was shown by rendering the CP inactive either by using C1q-depleted serum or by using an inhibitory antibody directed against the CP effector enzyme C1s. Reconstituting C1q-depleted serum with purified human C1q (10 μg/mL) restored CP-mediated C3b deposition (**Figures 3B, E**). In addition, inhibition of the CP using the anti-C1s inhibitory antibody TNT003 significantly reduced C3b deposition on the surface of *K. pneumoniae* and *S. aureus*. Under the chosen conditions, the residual C3b deposition is most likely the result of LP functional activity (**Figures 3C, F**).

### Secondary Loss of Complement Functional Activity Was Observed In Acute Severe COVID-19

We investigated the activity of the complement system in sera from acute and convalescent COVID-19 patients using a haemolytic assay with antibody-sensitised sheep erythrocytes. This assay provides an end-to-end measurement of complement activation *via* the CP and is sensitive to the reduction, absence and/or inactivity of any component of the CP and components involved in the formation of the lytic membrane attack complex.

**Figure 4:**
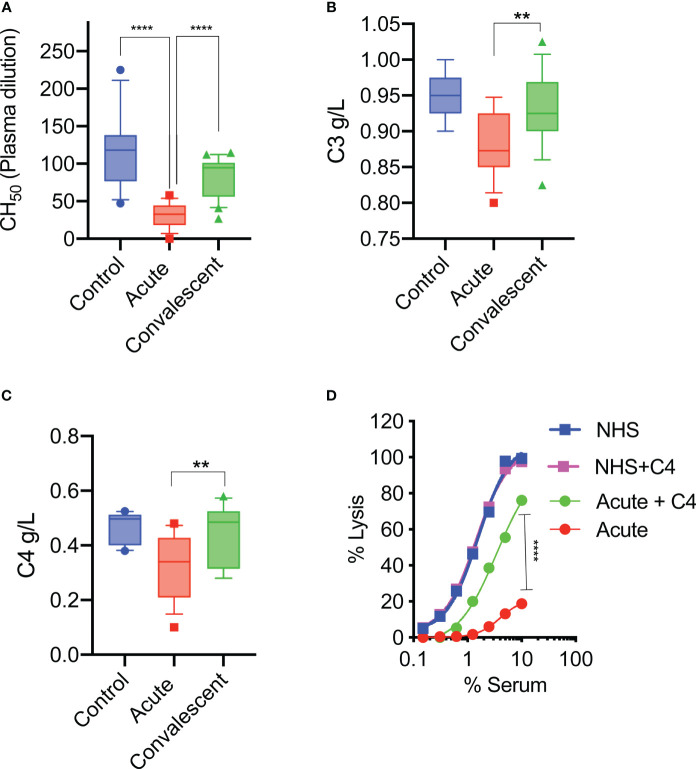
At hospital admission, sera of severely ill COVID-19 patients lack complement functional activity. Sheep RBCs were coated with anti-sheep erythrocyte antibodies and incubated with different serum concentrations. The serum dilution required to lyse 50% of RBCs (CH_50_) was calculated. The haemolytic activity of sera from acute COVID-19 patients (n= 25) is significantly impaired compared to sera from the same patients after recovery or to those of control NHS (n=14) **(A)**. Serum levels of complement C3 and C4 were also significantly lower in acute sera compared to convalescent sera and control sera (NHS) **(B, C)**. Results were analysed using 1-way ANOVA, with Dunnett’s correction for multiple comparisons. ****p < 0.0001, **p < 0.01. Reconstitution of sera taken during the acute phase of severe COVID-19 with 10 μg/mL of purified C4 restored the deficient haemolytic activity **(D)**. Results were analysed using 2-way ANOVA with Sidak’s multiple comparison test.

**Figure 5:**
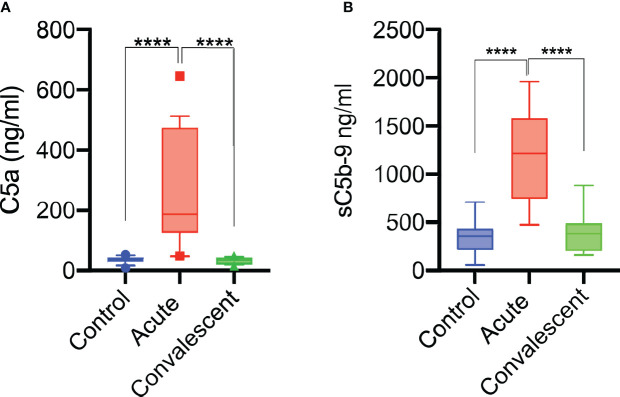
Complement system is activated during acute COVID-19 infection. Anaphylatoxin C5a levels in patients sera increase during acute disease and return to normal on recovery (n = 25) **(A)**. sC5b-9 levels were also significantly increased during the acute phase (n = 25) of severe COVID-19 and returned to levels seen in NHS (n = 14) of healthy blood donors and sera taken from convalescent patients **(B)**. Results were analysed using 1-way ANOVA, with Dunnett’s correction for multiple comparisons. ****p < 0.0001.

**Figure 6:**
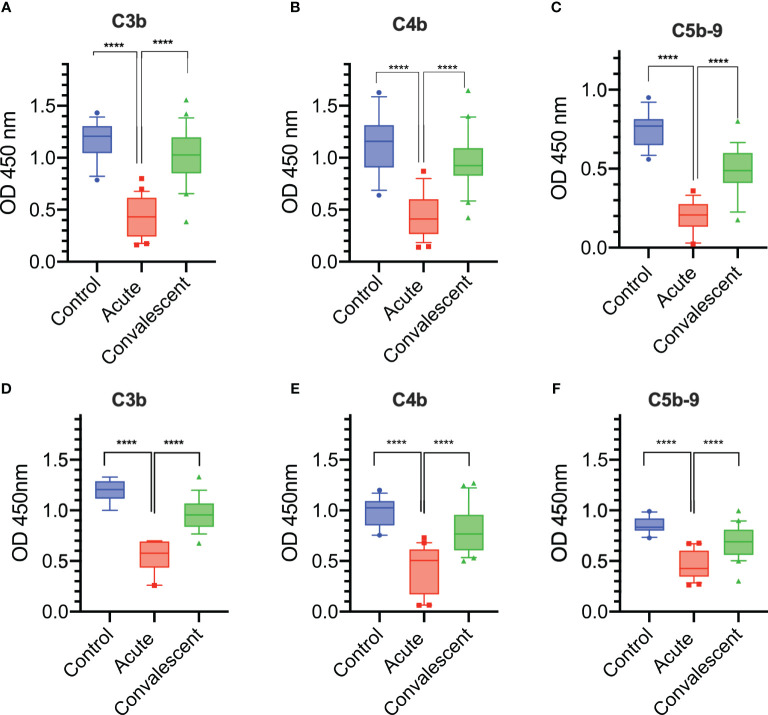
Complement deposition on the surface of *K. pneumoniae* and *S. aureus* is impaired during acute severe COVID-19. A significant reduction in C3b, C4b, and C5b-9 deposition levels on the surface of mucoid *K. pneumoniae* **(A–C)** and on the surface of *S. aureus* **(D–F)** were detected when using sera from acutely ill patients compared to sera from the same patients taken 3 months after discharge (n = 25) or to control sera ( n= 14). Results were analyzed using 1-way ANOVA, with Dunnett’s correction for multiple comparisons. ****p < 0.0001.

Sera were taken from 25 survivors of severe COVID-19 on admission to the ICU (acute sera) and 3 months after discharge (convalescent sera). All sera of patients with acute severe COVID-19 (on admission to ICU) showed little or no complement-mediated lysis, while convalescent sera from the same patients showed normal complement-mediated lysis 3 months after release from hospital (**Figure 4A**). The serum levels of C3 and C4 were also significantly lower in acute-phase sera compared to the convalescent sera of the same patients and significantly lower than in the NHS controls, supporting the hypothesis that acute-phase sera are hypocomplementaemic due to complement consumption in the early phase of severe COVID-19 (**Figures 4B, C**). Reconstitution of acute-phase sera with purified complement C4 restored the defective haemolytic activity, indicating that low levels of C4 in these sera at least contribute to defective CP and LP functional activity (**Figure 4D**). The hypothesis that hypocomplementaemia is resulting from hyperactivation of the complement system in the early phase of severe COVID-19 patients is supported by the detection of high levels of the complement activation markers C5a and sC5b-9 in acute patient sera compared to the levels seen in convalescent sera (**Figure 5**). To assess whether complement activation on the surface of *K. pneumoniae* is compromised during COVID-19 infection, we measured complement deposition on the surface of a mucoid strain (ATCC 43816) and a non-mucoid strain (Ecl8) using longitudinal serum samples of our study group of 25 patients with severe COVID-19. The levels of C3b, C4b and C5b-9 deposition were significantly lower in sera taken during the acute phase of the disease when compared to convalescent sera and NHS (**Figures 6A**–**C**). Similar results were obtained when the ELISA plates were coated with the non-mucoid strain Ecl8 (data not shown).

In addition, we assessed and compared complement deposition from acute and convalescent sera on the surface of *S. aureus* (the most common Gram-positive bacterium isolated in this study). As described for *K. pneumoniae*, all sera taken at the acute phase of severe COVID-19 were significantly compromised in their ability to deposit complement activation products, such as C3b, C4b and C5b-9, on the surface of *S. aureus* when compared to the degree of complement opsonisation seen in parallel when using NHS and convalescent sera of the same patients taken 3 months after release from hospital (**Figures 6D**–**F**).

### Serum Bactericidal Activity Against *K. pneumoniae* Is Impaired During Acute Severe COVID-19

**Figure 7:**
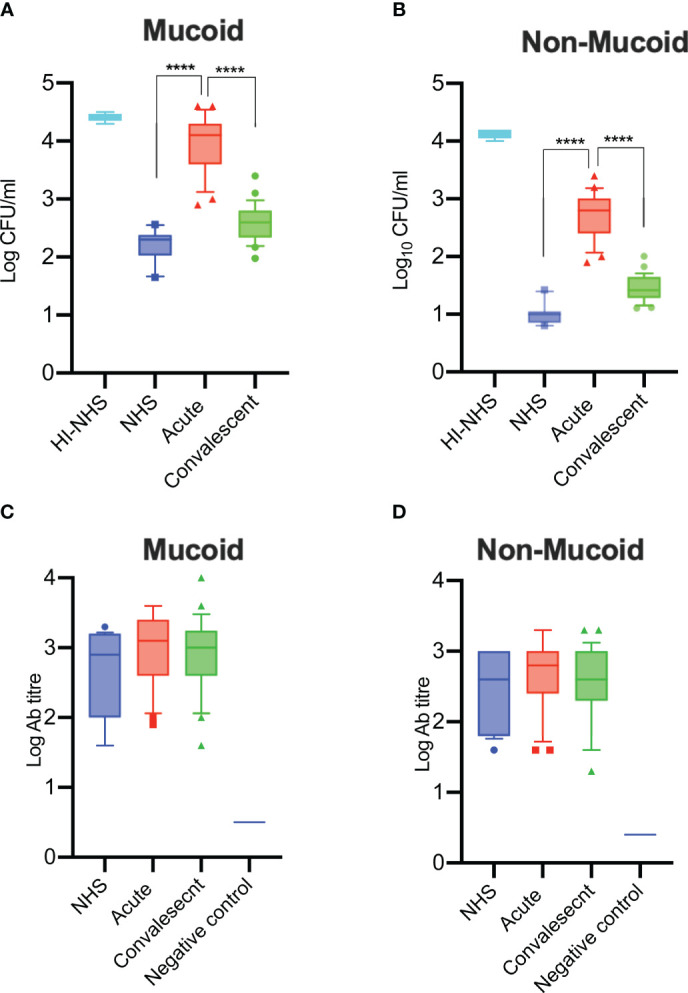
Serum bactericidal activity of sera from COVID-19 patients in the acute phase of severe disease is impaired and has recovered in convalescent sera. Complement-mediated killing of mucoid **(A)** and non-mucoid **(B)** strains of *K. pneumoniae* is significantly impaired in sera from acute patients compared to recovered patients in convalescence (n = 25 paired acute/convalescent patients). NHS (n = 12) and HI-NHS were used as controls. High antibody titres against *K. pneumoniae* species were detected in all 25 sera of patients with severe COVID-19 (C-D). An individual NHS sample with no detectable antibody titres against *K. pneumoniae* was used as a negative control **(C, D)**. Results were analysed using 1-way ANOVA, with Dunnett’s correction for multiple comparisons. ****p < 0.0001.

Having shown that serum from patients with acute severe COVID-19 is compromised in its ability to opsonise *K. pneumoniae* with complement C3b and C4b, we tested whether the bactericidal activity of the serum was similarly affected. Mucoid and non-mucoid strains of *K. pneumoniae* were incubated with sera from acute or convalescent patients and the number of viable bacteria after 2 hours was determined. Significantly lower levels of bacterial killing were observed when using sera from acute patients compared to sera from the same patients after recovery or to control NHS sera. Heat-inactivated serum (HI-NHS) was used as a negative control (**Figures 7A, B**). The presence of high antibody titres against mucoid and non-mucoid *K. pneumoniae* (**Figures 7C, D**) was also detected.

## Discussion

High rates of bacterial co-infection during previous outbreaks of pandemic and epidemic respiratory viral infection caused by H1N1, SARS-CoV-1 and MERS were associated with a high morbidity and mortality among infected patients (34, 35). Secondary infections with respiratory pathogens in COVID-19 patients have been reported in several previous studies (36–38). The upper respiratory tract hosts a wide range of commensal microorganisms, some of which are potential opportunistic pathogens including *Legionella pneumophila*, *Streptococcus pyogenes, Neisseria meningitidis, Moraxella catarrhalis, S. pneumoniae*, *H. influenzae*, *S. aureus*, *Pseudomonas aeruginosa* and *K. pneumoniae* (39). Infection with opportunistic bacteria and fungi in patients with severe COVID-19 is not surprising since studies from previous pandemic viral infections reported co-infection with opportunistic bacteria, fungi, and even other viruses (40).

It has been established that secondary bacterial infections in acute COVID-19 patients are associated with greater severity of COVID-19 and poorer outcome (41, 42) with a high incidence of Gram-negative infections, especially *K. pneumoniae* (43, 44). A high incidence of Gram-positive bacterial infections was also reported in COVID-19, mostly with *Staphylococcus species* (34). Co-infections with Gram-negative pathogens such as *Pseudomonas aeruginosa, Escherichia coli*, and enterobacteriaceae were also observed, yet *Klebsiella species* dominated and were identified in approximately 27% of patients infected with Gram-negative bacteria.

The prevalance of *Klebsiella* species was recently attributed to the ability of these bacteria to release several virulence factors that can overcome host immune defences, as well as the emergence of multi-drug-resistant strains of *K. pneumoniae* (45). The critical role of complement in fighting bacterial infections has been established in numerous animal studies as well as in clinical studies of inherited or acquired complement deficiencies (46, 47). Immune complex-mediated activation of the CP is recognised as an important mechanism in the control of *Klebsiella* infection (48, 49). In the presence of antibodies, *Klebsiella species* are generally kept in check by antibody and complement-mediated lysis and/or opsonophagocytosis (8), which makes them non-pathogenic commensals for most individuals, but the loss of complement functional activity poses a significantly increased risk for invasive infection.

Our present report showed a loss of complement functional activity in all acute-phase sera of patients assessed with severe COVID-19. Sera from these severely ill patients showed low CH_50_ and low levels of C3 and C4 (**Figure 4**). At the same time, complement activation products C5a and sC5b-9 were detected in abundance, suggesting that complement activation is a hallmark of the early phase of severe COVID-19 as previously postulated (50). Reconstitution of these sera with purified human C4 restored the defective complement-mediated haemolytic activity to levels seen in NHS. This supports the hypothesis that consumption of complement components during the early phase of severe COVID-19 leads to secondary hypocomplementaemia and impairs complement functional activity. The molecular events driving substantial complement activation in the early phase of severe COVID-19 are presently unknown. The reconstitution of haemolytic activity in acute-phase sera through the addition of purified C4 indicates involvement of either the lectin or the classical activation pathway, or both. A deficient alternative pathway functional activity seen in all acute-phase sera suggests that all 3 complement activation pathways may be involved. The high consumption of complement leading to hypocomplementaemia in blood taken at ICU admission implies that substantial complement activation occurs at an early phase of severe COVID-19, a time point that needs to be further defined.

A connection between complement activation and disease severity of COVID-19 was recently established by showing that the ratio between high levels of the complement activation product C3a and serum levels of C3 correlates with disease severity and might serve as a predictive marker of disease outcome (51, 52). Sinkovits et al. additionally described a low CH_50_ in sera of patients with severe COVID-19 (51). A loss of complement functional activity in sera of patients with severe COVID-19 has also been reported by Charitos et al. (53), showing a correlation between COVID-19 severity and a loss of complement functional activity *via* the classical and the alternative pathway. A study by Defendi et al. reported similar results demonstrating a significant reduction of total haemolytic activity in sera of a cohort of patients with severe COVID-19 compared to sera in a cohort of patients with a milder course of disease (54).

Whilst all patient sera in our study were positive for antibodies against *K. pneumoniae*, both SBA and complement opsonisation of *K. pneumoniae* (including deposition of C3b, C4b, and C5b-9) were compromised in sera of acute COVID-19 patients due to a loss of complement functional activity. This phenomenon is likely to increase the susceptibility not only to secondary infections with *K. pneumoniae* but also to other opportunistic pathogens that are usually held in check through complement-driven immune defense mechanisms.

In order to assess this, we measured complement deposition through each of the three complement activation pathways on a laboratory strain of *Staphylococcus aureus*, the most frequent Gram-positive opportunistic pathogen we detected in our group of severely ill COVID-19 patients. Again, complement deposition was highly impaired in sera of patients in the acute phase of severe COVID-19 and recovered in convalescent patient sera taken 3 months after hospital release. Acquired complement deficiency and low levels of C3, C4 and CH_50_ were previously reported to increase the risk of infection caused by *S. aureus* infection (55). A defect in complement opsonisation of *S. aureus* in sera of patients with acute severe COVID-19 may be a contributing factor to the previously reported high increase of nosocomial *Staphylococcus aureus* and *methicillin-resistant Staphylococcus aureus* (MRSA) infections in these patients (10).

## Conclusion

Our results support the hypothesis that secondary hypocomplementemia caused by complement consumption in the early phase of severe SARS-CoV-2 infection is a key risk factor for secondary microbial infections.

## Data Availability Statement

The original contributions presented in the study are included in the article/**Supplementary Material**. Further inquiries can be directed to the corresponding authors.

## Ethics Statement

The study was approved by Research Ethics Committee Wales, IRAS: 96194 12/WA/0148. Amendment 5. The patients/participants or their legal consent representatives provided written informed consent to participate in this study prior to enrolment.

## Author Contributions

YA, NL, SP, IEB, PK, AC, HB, and WS designed and performed the experiments. YA, NL, HB, and WS wrote and revised the manuscript. GD and MY provided essential reagents and revised the manuscript. YA and NL had full access to all data in the study, take responsibility for the integrity of the data, and affirm that the manuscript is an honest, accurate, and transparent account of the study being reported, and that no important aspects of the study have been omitted. All authors contributed to the article and approved the submitted version.

## Funding

This study was undertaken by HICC (Human Immune Correlates to COVID-19) consortium supported by NIHR/UKRI grant COV0170 awarded to WS.

## Conflict of Interest

WS, NL, YA are consultants to Omeros Corporation, which is developing inhibitors of the lectin pathway. GD and MY are employed by Omeros Corporation.

The remaining authors declare that the research was conducted in the absence of any commercial or financial relationships that could be construed as a potential conflict of interest.

## Publisher’s Note

All claims expressed in this article are solely those of the authors and do not necessarily represent those of their affiliated organizations, or those of the publisher, the editors and the reviewers. Any product that may be evaluated in this article, or claim that may be made by its manufacturer, is not guaranteed or endorsed by the publisher.

## Supplementary Material

Supplementary Figure 1 Heparin has no effect on complement activation at a concentration up to 10 IU/ml. NHS and heparinised plasma (10 IU/ml) or EDTA plasma (10 μM) were serially diluted in BBS^++^ and incubated in ELISA plates coated with *K. pneumoniae*. Complement C3b **(A)** and C4b **(B)** deposition were measured as described in materials and methods. No significant differences were observed in the degree of C3b and C4b deposition on *K. pneumoniae* when using NHS or plasma collected in heparin or EDTA tubes. The haemolytic activity of NHS and heparin or EDTA plasma was measured as described in materials and methods **(C)**. This assay provides an end-to-end measurement of complement activation *via* the CP, including components that are shared in all three pathways. No significant difference was observed in the haemolytic activity between serum and heparinised plasma used in this experiment.

## References

[1] Mizrahi B Shilo S Rossman H Kalkstein N Marcus K Barer Y. Longitudinal Symptom Dynamics of COVID-19 Infection. Nat Commun (2020) 11:6208. doi: 10.1038/s41467-020-20053-y PMC7718370 33277494

[2] Hughes S Troise O Donaldson H Mughal N Moore LSP. Bacterial and Fungal Coinfection Among Hospitalized Patients With COVID-19: A Retrospective Cohort Study in a UK Secondary-Care Setting. Clin Microbiol Infect (2020) 26:1395–9. doi: 10.1016/j.cmi.2020.06.025 PMC7320692 32603803

[3] Sharifipour E Shams S Esmkhani M Khodadadi J Fotouhi-Ardakani R Koohpaei A. Evaluation of Bacterial Co-Infections of the Respiratory Tract in COVID-19 Patients Admitted to ICU. BMC Infect Dis (2020) 20:646. doi: 10.1186/s12879-020-05374-z PMC7461753 32873235

[4] Han S Mallampalli RK. The Acute Respiratory Distress Syndrome: From Mechanism to Translation. J Immunol (2015) 194:855–60. doi: 10.4049/jimmunol.1402513 PMC4299926 25596299

[5] Martin RM Bachman MA. Colonization, Infection, and the Accessory Genome of Klebsiella Pneumoniae. Front Cell Infect Microbiol (2018) 8:4. doi: 10.3389/fcimb.2018.00004 29404282 PMC5786545

[6] Navon-Venezia S Kondratyeva K Carattoli A. Klebsiella Pneumoniae: A Major Worldwide Source and Shuttle for Antibiotic Resistance. FEMS Microbiol Rev (2017) 41:252–75. doi: 10.1093/femsre/fux013 28521338

[7] Lee HC Chuang YC Yu WL Lee NY Chang CM Ko NY. Clinical Implications of Hypermucoviscosity Phenotype in Klebsiella Pneumoniae Isolates: Association With Invasive Syndrome in Patients With Community-Acquired Bacteraemia. J Intern Med (2006) 259:606–14. doi: 10.1111/j.1365-2796.2006.01641.x 16704562

[8] Yu VL Hansen DS Ko WC Sagnimeni A Klugman KP von Gottberg A. Virulence Characteristics of Klebsiella and Clinical Manifestations of K. Pneumoniae Bloodstream Infections. Emerg Infect Dis (2007) 13:986–93. doi: 10.3201/eid1307.070187 PMC2878244 18214169

[9] Pollitt EJG Szkuta PT Burns N Foster SJ. Staphylococcus Aureus Infection Dynamics. PloS Pathog (2018) 14:e1007112. doi: 10.1371/journal.ppat.1007112 29902272 PMC6019756

[10] Adalbert JR Varshney K Tobin R Pajaro R. Clinical Outcomes in Patients Co-Infected With COVID-19 and Staphylococcus Aureus: A Scoping Review. BMC Infect Dis (2021) 21:985. doi: 10.1186/s12879-021-06616-4 PMC8453255 34548027

[11] Cusumano JA Dupper AC Malik Y Gavioli EM Banga J Berbel Caban A. Staphylococcus Aureus Bacteremia in Patients Infected With COVID-19: A Case Series. Open Forum Infect Dis (2020) 7:ofaa518. doi: 10.1093/ofid/ofaa518 33269299 PMC7686656

[12] Heesterbeek DAC Martin NI Velthuizen A Duijst M Ruyken M Wubbolts R. Complement-Dependent Outer Membrane Perturbation Sensitizes Gram-Negative Bacteria to Gram-Positive Specific Antibiotics. Sci Rep (2019) 9:3074. doi: 10.1038/s41598-019-38577-9 PMC6395757 30816122

[13] Schwaeble W Dahl MR Thiel S Stover C Jensenius JC. The Mannan-Binding Lectin-Associated Serine Proteases (MASPs) and MAp19: Four Components of the Lectin Pathway Activation Complex Encoded by Two Genes. Immunobiology (2002) 205:455–66. doi: 10.1078/0171-2985-00146 12396007

[14] Lachmann PJ. The Amplification Loop of the Complement Pathways. Adv Immunol (2009) 104:115–49. doi: 10.1016/S0065-2776(08)04004-2 20457117

[15] Ali YM Hayat A Saeed BM Haleem KS Alshamrani S Kenawy HI. Low-Dose Recombinant Properdin Provides Substantial Protection Against Streptococcus Pneumoniae and Neisseria Meningitidis Infection. Proc Natl Acad Sci USA (2014) 111:5301–6. doi: 10.1073/pnas.1401011111 PMC3986141 24706855

[16] Gralinski LE Sheahan TP Morrison TE Menachery VD Jensen K Leist SR. Complement Activation Contributes to Severe Acute Respiratory Syndrome Coronavirus Pathogenesis. mBio (2018) 9. doi: 10.1128/mBio.01753-18 PMC6178621 30301856

[17] Magro C Mulvey JJ Berlin D Nuovo G Salvatore S Harp J. Complement Associated Microvascular Injury and Thrombosis in the Pathogenesis of Severe COVID-19 Infection: A Report of Five Cases. Transl Res (2020) 220:1–13. doi: 10.1016/j.trsl.2020.04.007 32299776 PMC7158248

[18] Java A Apicelli AJ Liszewski MK Coler-Reilly A Atkinson JP Kim AH. The Complement System in COVID-19: Friend and Foe? JCI Insight (2020) 5. doi: 10.1172/jci.insight.140711 PMC7455060 32554923

[19] Agostinis C Mangogna A Balduit A Aghamajidi A Ricci G Kishore U. COVID-19, Pre-Eclampsia, and Complement System. Front Immunol (2021) 12:775168. doi: 10.3389/fimmu.2021.775168 34868042 PMC8635918

[20] Jensen TS Opstrup KV Christiansen G Rasmussen PV Thomsen ME Justesen DL. Complement Mediated Klebsiella Pneumoniae Capsule Changes. Microbes Infect (2020) 22:19–30. doi: 10.1016/j.micinf.2019.08.003 31473336

[21] van der Maten E de Jonge MI de Groot R van der Flier M Langereis JD. A Versatile Assay to Determine Bacterial and Host Factors Contributing to Opsonophagocytotic Killing in Hirudin-Anticoagulated Whole Blood. Sci Rep (2017) 7:42137. doi: 10.1038/srep42137 28176849 PMC5296863

[22] Doorduijn DJ Bardoel BW Heesterbeek DAC Ruyken M Benn G Parsons ES. Bacterial Killing by Complement Requires Direct Anchoring of Membrane Attack Complex Precursor C5b-7. PloS Pathog (2020) 16:e1008606. doi: 10.1371/journal.ppat.1008606 32569291 PMC7351214

[23] Abu-Humaidan AH Elven M Sonesson A Garred P Sorensen OE. Persistent Intracellular Staphylococcus Aureus in Keratinocytes Lead to Activation of the Complement System With Subsequent Reduction in the Intracellular Bacterial Load. Front Immunol (2018) 9:396. doi: 10.3389/fimmu.2018.00396 29545804 PMC5837974

[24] Cui S Chen S Li X Liu S Wang F. Prevalence of Venous Thromboembolism in Patients With Severe Novel Coronavirus Pneumonia. J Thromb Haemost (2020) 18:1421–4. doi: 10.1111/jth.14830 PMC7262324 32271988

[25] Klok FA Kruip MJHA van der Meer NJM Arbous MS Gommers D A M P J Kant KM. Incidence of Thrombotic Complications in Critically Ill ICU Patients With COVID-19. Thromb Res (2020) 191:145–7. doi: 10.1016/j.thromres.2020.04.013 PMC7146714 32291094

[26] DeLeo FR Kobayashi SD Porter AR Freedman B Dorward DW Chen L. Survival of Carbapenem-Resistant Klebsiella Pneumoniae Sequence Type 258 in Human Blood. Antimicrob Agents Chemother (2017) 61. doi: 10.1128/AAC.02533-16 PMC5365663 28115349

[27] Sahu A Morikis D Lambris JD. Compstatin, a Peptide Inhibitor of Complement, Exhibits Species-Specific Binding to Complement Component C3. Mol Immunol (2003) 39:557–66. doi: 10.1016/S0161-5890(02)00212-2 12431389

[28] Dodds AW. Small-Scale Preparation of Complement Components C3 and C4. Methods Enzymol (1993) 223:46–61. doi: 10.1016/0076-6879(93)23037-N 8271967

[29] Petersen SV Thiel S Jensen L Steffensen R Jensenius JC. An Assay for the Mannan-Binding Lectin Pathway of Complement Activation. J Immunol Methods (2001) 257:107–16. doi: 10.1016/S0022-1759(01)00453-7 11687244

[30] Peerschke EI Panicker S Bussel J. Classical Complement Pathway Activation in Immune Thrombocytopenia Purpura: Inhibition by a Novel C1s Inhibitor. Br J Haematol (2016) 173:942–5. doi: 10.1111/bjh.13648 PMC4973859 26305671

[31] Kenawy HI Ali YM Rajakumar K Lynch NJ Kadioglu A Stover CM. Absence of the Lectin Activation Pathway of Complement Does Not Increase Susceptibility to Pseudomonas Aeruginosa Infections. Immunobiology (2012) 217:272–80. doi: 10.1016/j.imbio.2011.10.001 22070931

[32] Bidmos FA Chan H Praekelt U Tauseef I Ali YM Kaczmarski EB. Investigation Into the Antigenic Properties and Contributions to Growth in Blood of the Meningococcal Haemoglobin Receptors, HpuAB and HmbR. PloS One (2015) 10:e0133855. doi: 10.1371/journal.pone.0133855 26208277 PMC4514712

[33] Lundberg U Senn BM Schuler W Meinke A Hanner M. Identification and Characterization of Antigens as Vaccine Candidates Against Klebsiella Pneumoniae. Hum Vaccin Immunother (2013) 9:497–505. doi: 10.4161/hv.23225 23250007 PMC3891705

[34] Langford BJ So M Raybardhan S Leung V Westwood D MacFadden DR. Bacterial Co-Infection and Secondary Infection in Patients With COVID-19: A Living Rapid Review and Meta-Analysis. Clin Microbiol Infect (2020) 26:1622–9. doi: 10.1016/j.cmi.2020.07.016 PMC7832079 32711058

[35] Joseph C Togawa Y Shindo N. Bacterial and Viral Infections Associated With Influenza. Influenza Other Respir Viruses (2013) 7 Suppl 2:105–13. doi: 10.1111/irv.12089 PMC5909385 24034494

[36] Yang X Yu Y Xu J Shu H Xia J Liu H. Clinical Course and Outcomes of Critically Ill Patients With SARS-CoV-2 Pneumonia in Wuhan, China: A Single-Centered, Retrospective, Observational Study. Lancet Respir Med (2020) 8:475–81. doi: 10.1016/S2213-2600(20)30079-5 PMC7102538 32105632

[37] Russell CD Fairfield CJ Drake TM Turtle L Seaton RA Wootton DG. Co-Infections, Secondary Infections, and Antimicrobial Use in Patients Hospitalised With COVID-19 During the First Pandemic Wave From the ISARIC WHO CCP-UK Study: A Multicentre, Prospective Cohort Study. Lancet Microbe (2021) 2:e354–65. doi: 10.1016/S2666-5247(21)00090-2 PMC8172149 34100002

[38] He S Liu W Jiang M Huang P Xiang Z Deng D. Clinical Characteristics of COVID-19 Patients With Clinically Diagnosed Bacterial Co-Infection: A Multi-Center Study. PloS One (2021) 16:e0249668. doi: 10.1371/journal.pone.0249668 33819304 PMC8021165

[39] Morris DE Cleary DW Clarke SC. Secondary Bacterial Infections Associated With Influenza Pandemics. Front Microbiol (2017) 8:1041. doi: 10.3389/fmicb.2017.01041 28690590 PMC5481322

[40] Frias-De-Leon MG Pinto-Almazan R Hernandez-Castro R Garcia-Salazar E Meza-Meneses P Rodriguez-Cerdeira C. Epidemiology of Systemic Mycoses in the COVID-19 Pandemic. J Fungi (Basel) (2021) 7. doi: 10.3390/jof7070556 PMC8307417 34356935

[41] Farrell JM Zhao CY Tarquinio KM Brown SP. Causes and Consequences of COVID-19-Associated Bacterial Infections. Front Microbiol (2021) 12:682571. doi: 10.3389/fmicb.2021.682571 34354682 PMC8329088

[42] Feldman C Anderson R. The Role of Co-Infections and Secondary Infections in Patients With COVID-19. Pneumonia (Nathan) (2021) 13:5–021. doi: 10.1186/s41479-021-00083-w 33894790 PMC8068564

[43] Emeraud C Figueiredo S Bonnin RA Khecharem M Ouzani S Leblanc P. Outbreak of CTX-M-15 Extended-Spectrum β-Lactamase-Producing *Klebsiella Pneumoniae* ST394 in a French Intensive Care Unit Dedicated to COVID-19. Pathogens (2021) 10:1426. doi: 10.3390/pathogens10111426 34832582 PMC8618658

[44] Mędrzycka-Dąbrowska W Lange S Zorena K Dąbrowski S Ozga D Tomaszek L. Carbapenem-Resistant *Klebsiella Pneumoniae* Infections in ICU COVID-19 Patients—A Scoping Review. J Clin Med (2021) 10:2067. doi: 10.3390/jcm10102067 34066031 PMC8150615

[45] Kim D Park BY Choi MH Yoon EJ Lee H Lee KJ. Antimicrobial Resistance and Virulence Factors of Klebsiella Pneumoniae Affecting 30 Day Mortality in Patients With Bloodstream Infection. J Antimicrob Chemother (2019) 74:190–9. doi: 10.1093/jac/dky397 30295771

[46] Brown EJ Hosea SW Hammer CH Burch CG Frank MM. A Quantitative Analysis of the Interactions of Antipneumococcal Antibody and Complement in Experimental Pneumococcal Bacteremia. J Clin Invest (1982) 69:85–98. doi: 10.1172/jci110444 7054244 PMC371171

[47] Bousfiha A Jeddane L Picard C Ailal F Bobby Gaspar H Al-Herz W. The 2017 IUIS Phenotypic Classification for Primary Immunodeficiencies. J Clin Immunol (2018) 38:129–43. doi: 10.1007/s10875-017-0465-8 PMC5742599 29226301

[48] Bengoechea JA Sa Pessoa J. Klebsiella Pneumoniae Infection Biology: Living to Counteract Host Defences. FEMS Microbiol Rev (2019) 43:123–44. doi: 10.1093/femsre/fuy043 PMC6435446 30452654

[49] Alberti S Marques G Hernandez-Alles S Rubires X Tomas JM Vivanco F. Interaction Between Complement Subcomponent C1q and the Klebsiella Pneumoniae Porin Ompk36. Infect Immun (1996) 64:4719–25. doi: 10.1128/iai.64.11.4719-4725.1996 PMC174437 8890231

[50] Ma L Sahu SK Cano M Kuppuswamy V Bajwa J McPhatter J. Increased Complement Activation Is a Distinctive Feature of Severe SARS-CoV-2 Infection. Sci Immunol (2021) 6. doi: 10.1126/sciimmunol.abh2259 PMC8158979 34446527

[51] Sinkovits G Mezo B Reti M Muller V Ivanyi Z Gal J. Complement Overactivation and Consumption Predicts In-Hospital Mortality in SARS-CoV-2 Infection. Front Immunol (2021) 12:663187. doi: 10.3389/fimmu.2021.663187 33841446 PMC8027327

[52] de Nooijer AH Grondman I Janssen NAF Netea MG Willems L van de Veerdonk FL. Complement Activation in the Disease Course of Coronavirus Disease 2019 and Its Effects on Clinical Outcomes. J Infect Dis (2021) 223:214–24. doi: 10.1093/infdis/jiaa646 PMC7797765 33038254

[53] Charitos P Heijnen IAFM Egli A Bassetti S Trendelenburg M Osthoff M. Functional Activity of the Complement System in Hospitalized COVID-19 Patients: A Prospective Cohort Study. Front Immunol (2021) 12:765330. doi: 10.3389/fimmu.2021.765330 34777382 PMC8581394

[54] Defendi F Leroy C Epaulard O Clavarino G Vilotitch A Le Marechal M. Complement Alternative and Mannose-Binding Lectin Pathway Activation Is Associated With COVID-19 Mortality. Front Immunol (2021) 12:742446. doi: 10.3389/fimmu.2021.742446 34567008 PMC8461024

[55] Homann C Varming K Hogasen K Mollnes TE Graudal N Thomsen AC. Acquired C3 Deficiency in Patients With Alcoholic Cirrhosis Predisposes to Infection and Increased Mortality. Gut (1997) 40:544–9. doi: 10.1136/gut.40.4.544 PMC1027133 9176087

