# Supplementary material for: Secondary Complement Deficiency Impairs Anti-Microbial Immunity to Klebsiella pneumoniae and Staphylococcus aureus During Severe Acute COVID-19
Source: Front Immunol. 2022 Apr 27;13:841759. doi: 10.3389/fimmu.2022.841759 (PMC9094484; doi:10.3389/fimmu.2022.841759)
Supplement: Supplementary Figure 1 — Heparin has no effect on complement activation at a concentration up to 10 IU/ml. NHS and heparinised plasma (10 IU/ml) or EDTA plasma (10 μM) were serially diluted in BBS++ and incubated in ELISA plates coated with K. pneumoniae. Complement C3b (A) and C4b (B) deposition were measured as described in materials and methods. No significant differences were observed in the degree of C3b and C4b deposition on K. pneumoniae when using NHS or plasma collected in heparin or EDTA tubes. The haemolytic activity of NHS and heparin or EDTA plasma was measured as described in materials and methods (C). This assay provides an end-to-end measurement of complement activation via the CP, including components that are shared in all three pathways. No significant difference was observed in the haemolytic activity between serum and heparinised plasma used in this experiment. [file DataSheet_1.docx]

**Supporting information**

**S1: Heparin has no effect on complement activation at a concentration up to 10 IU/ml**. NHS and heparinised plasma (10 IU/ml) or EDTA plasma (10 μM) were serially diluted in BBS^++^ and incubated in ELISA plates coated with *K. pneumoniae*. Complement C3b (A) and C4b (B) deposition were measured as described in materials and methods. No significant differences were observed in the degree of C3b and C4b deposition on *K. pneumoniae* when using NHS or plasma collected in heparin or EDTA tubes. The haemolytic activity of NHS and heparin or EDTA plasma was measured as described in materials and methods (C). This assay provides an end-to-end measurement of complement activation via the CP, including components that are shared in all three pathways. No significant difference was observed in the haemolytic activity between serum and heparinised plasma used in this experiment.
